# Total management of hemangiopericytoma/solitary fibrous tumor of the buttock: A case report

**DOI:** 10.1097/MD.0000000000039044

**Published:** 2024-07-19

**Authors:** Kazuhiko Hashimoto, Shunji Nishimura, Tomohiko Ito, Ryosuke Kakinoki, Koji Goto

**Affiliations:** a Department of Orthopedic Surgery, Kindai University Hospital, Osaka-Sayama City, Osaka, Japan.

**Keywords:** buttock, case report, hemangiopericytoma, management, solitary fibrous tumor

## Abstract

**Background:**

Solitary fibrous tumors can manifest at various anatomical sites, predominantly occurring at extrapleural sites with a peak incidence between 40 and 70 years. SFT necessitates long-term follow-up owing to its tumor characteristics. However, comprehensive reports covering the period from initial diagnosis to the patient’s demise are lacking. Herein, we present a case of a malignant SFT of the buttocks that was treated at our hospital from the time of initial diagnosis to the end of life, with a literature review.

**Methods:**

A 54-year-old woman had a T1 low-to-isobaric and T2 isobaric-to-hyperintense mass in the psoas muscle on magnetic resonance imaging, diagnosed as an SFT. Wide excision was performed, followed by postoperative radiotherapy and chemotherapy. Multiple lung metastases were treated, while bone metastases appeared in the left femur. Multiple spinal metastases developed, causing respiratory distress due to pleural effusion. Best support care was initiated; however, a thrombus appeared in the inferior vena cava. Despite anticoagulant therapy, the patient died 11 years and 6 months after the initial surgery. Herein, marginal resection resulted in a relatively short operative time and average blood loss. The radiotherapy dose was 66 Gy; no complications occurred, and local recurrence was prevented. Tumor arthroplasty was performed to stabilize the affected limbs, and the patient required careful follow-up.

**Results:**

Despite the poor prognosis, the patient survived >11 years after surgery and had a favorable outcome.

**Conclusion:**

Long-term monitoring for potential complications remains necessary.

## 1. Introduction

A solitary fibrous tumor (SFT) was identified as the same entity as hemangiopericytoma per the World Health Organization (WHO) classification in 2016.^[1]^ SFTs can occur anywhere in the body.^[1]^ They are not differentiated by sex and are more common in individuals aged 40 to 70 years.^[1]^ Approximately 30% to 40% of SFTs occur in the pleura, with a similar percentage occurring in the extremities, deep soft tissues, abdominal cavity, pelvis, or retroperitoneum.^[1–3]^ Deep-seated tumors are more common than superficial tumors, accounting for 70% to 90% of cases.^[1]^ Typically, SFTs are slow-growing and painless.^[1]^ Abdominopelvic SFTs may present with symptoms like distention, constipation, urinary retention, or early satiety, whereas head and neck SFTs may present with nasal obstruction, voice changes, or bleeding.^[1]^ The radiographic features of SFT are largely nonspecific.^[1,4]^ Moreover, the genetic hallmark of SFT is characterized by the involvement of chromosome 12q, resulting in the fusion of the NAB2 and STAT6 genes.^[1,2]^ SFTs are well-defined masses measuring 5 to 10 cm, although some lesions may exceed 25 cm or greater in dimension.^[1]^ Histologically, SFTs are variable cell tumors composed of oval to spindle-shaped cells that exhibit an unpatterned growth or storiform pattern against a variable collagenous background stroma containing thin-walled, large, branching “staghorn” type (HPC-like) vessels.^[3]^ In addition, malignant peripheral nerve sheath tumors are composed of cell sheets of spindle cells that may alternate with hypocellular areas. Tumor cells may also exhibit HPC-like vasculature and enhancement around blood vessels, making it difficult to make a differential diagnosis from SFT.^[3]^ SFTs also recur and metastasize even 10 years after the initial surgery.^[1]^ Despite several case reports and literature reviews regarding SFT,^[2–4]^ the course of this case has been reported from the initial diagnosis to the end of life. In addition, no case reports of treatment exist across WHO classification revisions. The purpose of this case report is to present a case of a malignant soft tissue tumor of the buttocks that was treated at our hospital from the time of initial diagnosis to the end of life, with a literature review. This report constitutes the first complete record of SFT treatment. Thus, the details of this comprehensive SFT treatment record will benefit oncologic surgeons worldwide who encounter challenges in treating SFT.

## 2. Case Report

**Figure 1.**
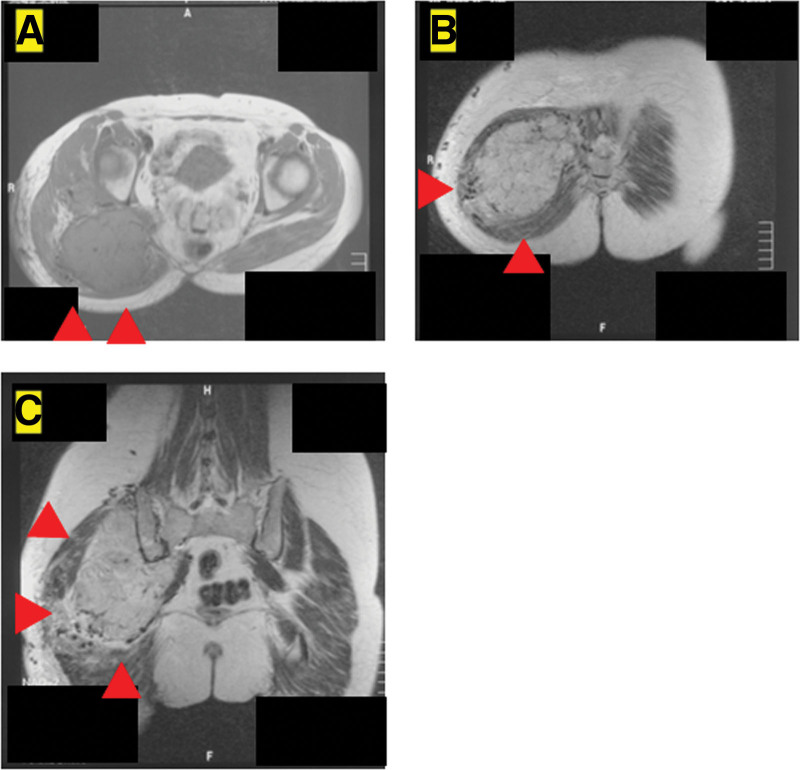
Axial T1-weighted (A) and T2-weighted (B) magnetic resonance imaging (MRI) of the buttocks. Coronal T2-weighted image (C) of the buttock. The red arrowheads indicate the tumor mass.

A 54-year-old woman initially presented with a lump on her right buttock for 3 years before she visited the orthopedic surgery department of Kindai University Hospital (August 1, 2009). She had no pain and chose to ignore the lump. The lump gradually enlarged, and the patient visited her local doctor, who referred her to our department. Notably, her medical records indicated no family or personal history of thrombosis. Blood samples collected during the visit showed no obvious abnormalities (Table 1). Clinical examination and magnetic resonance imaging (MRI) revealed a 26 × 20 cm tumor with no tenderness, Tinel sign, or elastic firmness but had poor elasticity, resulting in poor mobility (Fig. 1A–C). MRI showed a T1 low-to-isobaric and T2 isobaric-to-hyperintense mass deep in the psoas muscle (Fig. 1A–C). Subsequently, a needle biopsy was performed, and tissue samples were obtained from within the tumor. Hematoxylin and eosin (H&E) staining was performed on the collected tissue specimens. Following deparaffinization and dehydration with alcohol for 1 minute, the specimens were stained with hematoxylin for 10 minutes to visualize cell nuclei, resulting in a blue–purple hue. Subsequently, eosin staining was performed for 5 minutes to highlight cytoplasmic structures. After another round of alcohol dehydration for 1 minute, the specimens were immersed in xylene for 5 minutes. Additional immunostaining for CD34, Factor VIII, S-100, and smooth muscle actin (SMA) was carried out. The specimens underwent paraffin removal with xylene or ethanol for 5 minutes, followed by antigen activation through heating to 37°C. Finally, endogenous peroxidase in the tissue was neutralized.

**Table 1:** Preoperative blood test results.

Item	Value	Lower limit	Upper limit
CRP (mg/dL)	0.189	0	0.14
Cr (mg/dL)	0.58	0.46	0.79
Alb (g/dL)	4.8	4.1	5.1
AST (U/L)	21	13	30
ALT (U/L)	20	7	23
Leukocytes (×10^3^/µL)	4.80	3.3	8.6
Hb (g/dL)	13.0	11.6	14.8
PLT (×10^4^/µL)	20.8	15.8	34.8
Neutrophils (×10^3^/µL)	3.02	4.0	8.0

Alb = albumin, ALT = alanine aminotransferase, AST = aspartate aminotransferase, Cr = creatinine, CRP = C-reactive protein, Hb = hemoglobin, PLT = platelets.

**Figure 2.**
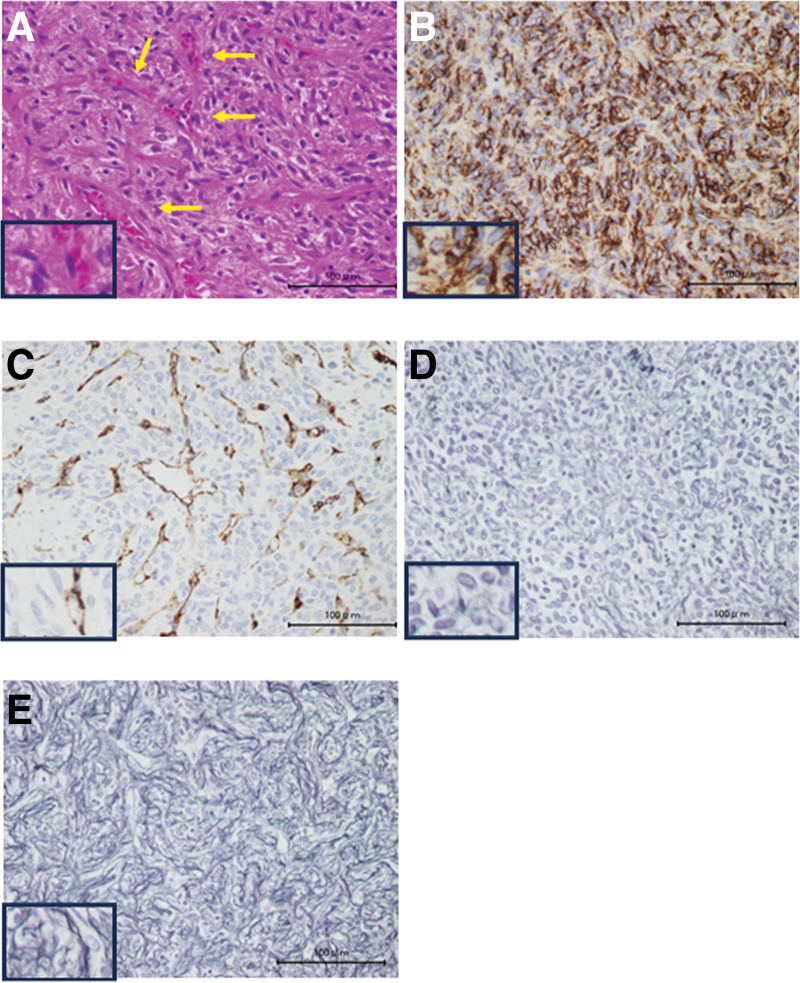
Hematoxylin and eosin (HE) staining of the specimen (A). An increase in spindle-shaped cells with nuclear atypia and blood vessels was observed. Immunostaining of the specimens for CD34+ (B). Immunostaining of the specimens for Factor VIII (C). Scale bar = 100 µm. Immunostaining of the specimens for S-100 and SMA (D, E). The image encircled by the square in the lower left corner of each is enlarged.

Primary antibodies were applied and allowed to incubate for 10 minutes as follows: for CD34; Anti-CD34 [EP373Y] (ab81289; 1:100 solution), Factor VIII; Anti-Factor VIII antibody [GMA-012] (ab78852; 1:200 solution), S-100; Anti-S100 (ab34686; 1:100 solution), and SMA; ACTA2/SMA antibody (Anti ACTA2/SMA antibody; 1:200 solution; Funakoshi). After washing, the secondary antibodies were applied for 20 minutes each: (for CD34: Goat Anti-Rabbit IgG H&L horseradish peroxidase [HRP] preadsorbed [ab7090; 1:200 solution], Factor VIII: Goat Anti-Mouse IgG + IgM H&L HRP preadsorbed [ab47827; 1:100 solution], S-100: Goat Anti-Rabbit IgG H&L HRP preadsorbed [ab7090; 1:200 solution], and SMA: Goat Anti-Mouse IgG + IgM H&L HRP preadsorbed [ab47827; 1:100 solution]). Subsequently, counterstaining was performed with a hematoxylin solution for 5 minutes. After another washing step, permeabilization was carried out with alcohol. Histological findings revealed an increase in the number of spindle-shaped cells with nuclear atypia (Fig. 2A). These cells were particularly abundant in the blood vessels (Fig. 2A). Vascular tissue identification was confirmed by staining the vessel walls and lumen with CD34 and Factor VIII. The immunostaining result showed positivity for CD34 (Fig. 2B) and Factor VIII (Fig. 2C) but negativity for S-100 and SMA (Figures 2D and E).

**Figure 3.**
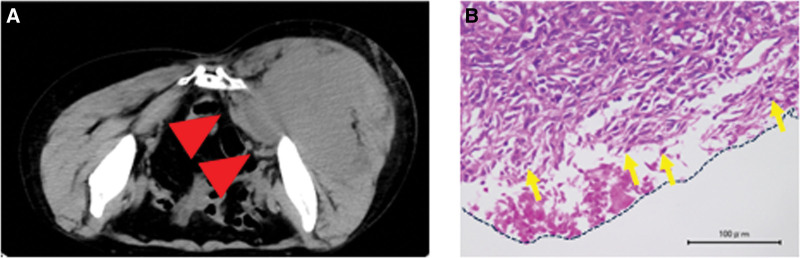
Computed tomography (CT) axial images show tumor encroachment into the pelvis (A, red arrowheads). H&E stained image of the resected specimen with positive surgical margins (B, yellow arrows indicate tumor cells, and the surgical margin is delineated by the black broken line).

The initial differential diagnosis, including malignant peripheral schwannoma and synovial sarcoma, were ruled out as they tested negative for S-100 and SMA, respectively. SFT was initially suspected due to the dense proliferation of spindle-shaped atypical cells with unequally sized and unequal round nuclei. The final diagnosis was confirmed by the presence of scattered vascular spaces and immunostaining for cytokeratin, S-100 (−), and SMA (−). Subsequently, the patient was diagnosed with an SFT, and a wide excision was performed on August 26, 2009. Preoperative images showed that the tumor extended into the pelvis through the sciatic notch (Fig. 3A); therefore, preoperative embolization was attempted but abandoned owing to the risk of lower extremity gangrene. Partial marginal excision was performed. The sciatic nerve and incisions were treated with alcohol. Operative time was 2 hours 3 minutes, and blood loss was 518 mL.

**Figure 4.**
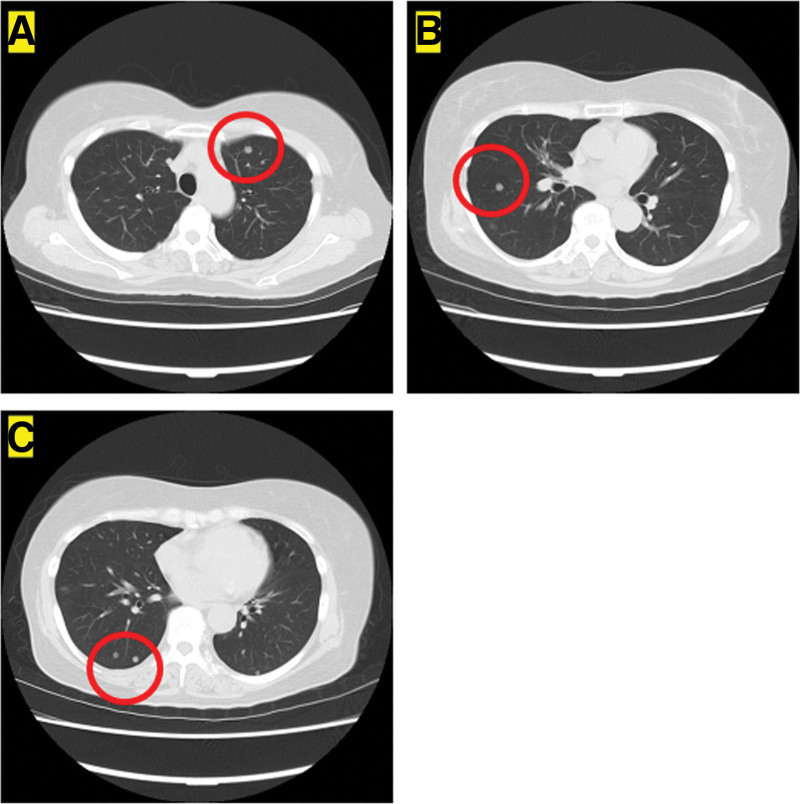
Axial CT image of the lung (A–C). Multiple metastases are observed (A–C). The red circle indicates the metastasis region.

Postoperative margins were positive (Fig. 3B), as confirmed by the presence of tumor cells at the resection margin in the pathology specimen. Subsequently, postoperative radiation therapy (RT) was administered by a radiologist (66 Gy/33 Fr). This decision was based on evidence indicating that adjuvant RT may obviate the need for re-resection for selected patients with positive or close pathologic margins in managing extremity and truncal patients with soft tissue sarcomas (STS).^[5]^ The oncologist started weekly paclitaxel (PTX) as postoperative chemotherapy. This choice was informed by evidence suggesting the effectiveness of weekly PTX, cisplatin, trastuzumab, and cranial radiotherapy as adjuvant therapy for hemangiopericytoma.^[6,7]^ Five months later (February 14, 2010), the patient developed multiple lung metastases (Fig. 4A–C). Weekly PTX proved ineffective, and the patient was diagnosed with progressive disease. Subsequently, pazopanib (an antivascular endothelial growth factor drug) was administered at a dose of 200 mg/day, based on existing evidence indicating a 79% partial response and 14% stable disease in patients with hemangiopericytoma/malignant SFT if treated with daily pazopanib.^[8]^ The decision to start with a lower dose was made considering the patient’s relatively advanced age and the presence of a pleural effusion, which may have rendered the full dose of 800 mg/day intolerable owing to potential side effects.

Chemotherapy was administered in the hospital, during which the patient experienced a fever of approximately 37.5°C; however, no significant elevation was observed in C-reactive protein levels.

**Figure 5.**
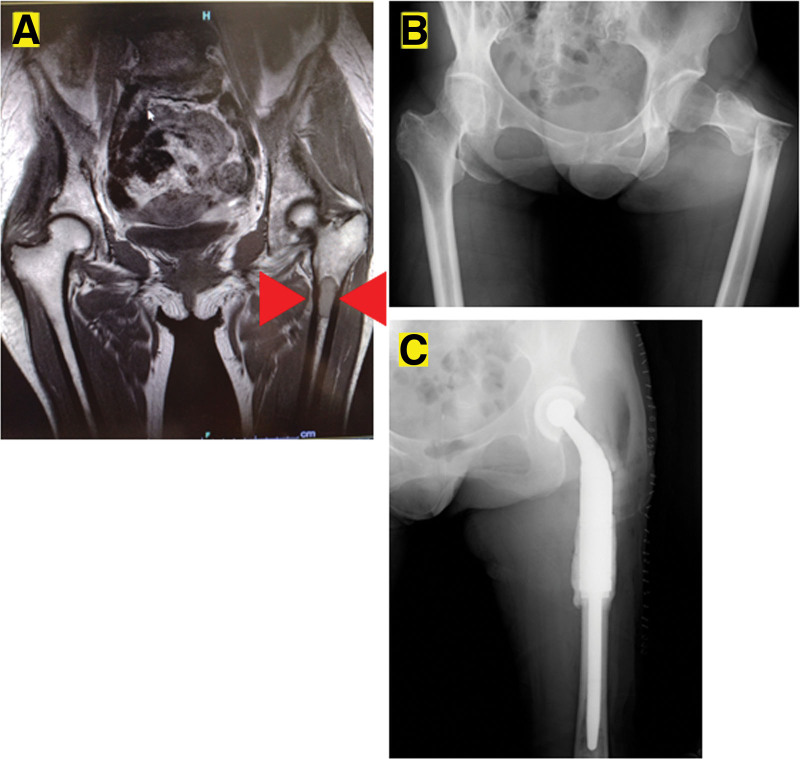
Magnetic resonance image of the hip (A). Metastasis region of the left femur is observed (A). Radiograph of the hip (B). The pathological fracture is observed (B). Radiograph of the left femur after surgical treatment with endoprosthesis (C).

**Figure 6.**
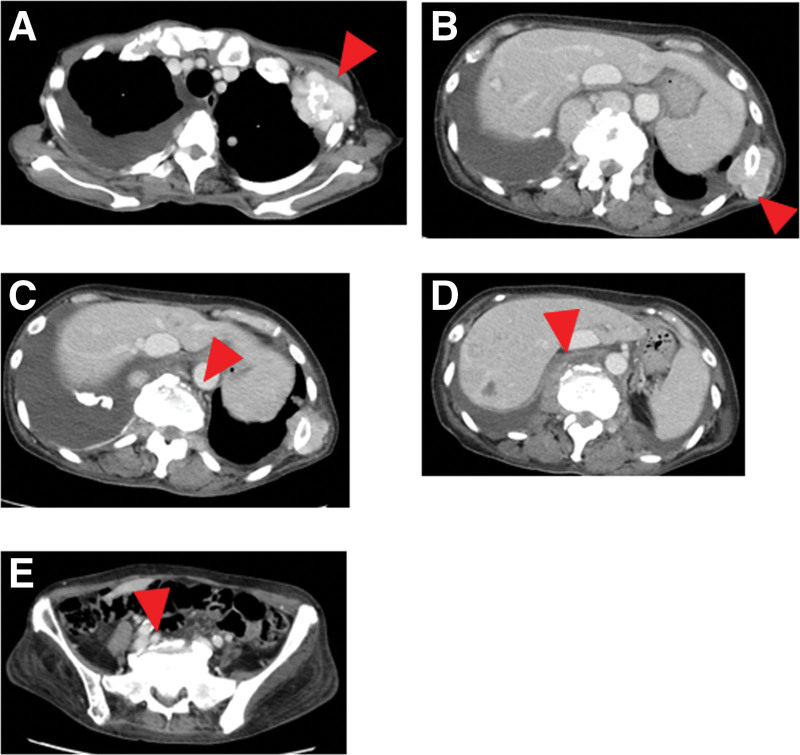
CT axial images reveal the metastatic lesion in the second rib (A, red arrowhead), tenth rib (B, red arrowhead), and multiple spinal metastatic lesions (C–E, red arrowheads).

**Figure 7.**
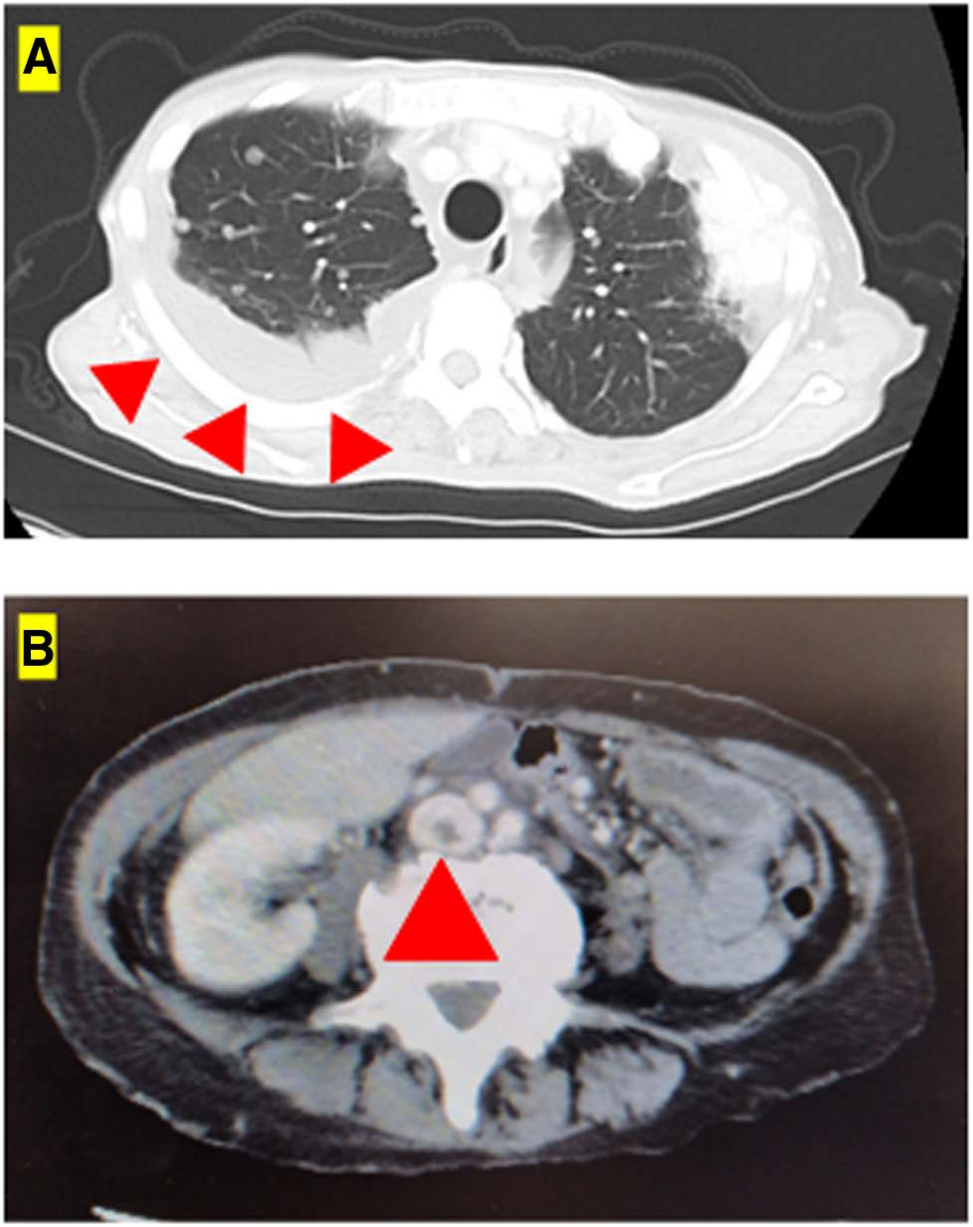
CT axial images show pleural effusion in the right lung field (A; red arrowheads) and thrombus in the inferior vena cava (B; red arrowhead).

The number of pulmonary metastases did not increase. Pazopanib was continued, and the disease was stable. Seven years after the surgery, bone metastasis was observed in the left femur (Fig. 5A) on October 13, 2016. Despite the appearance of bone metastases, treatment with pazopanib was maintained. Six months later, a pathological fracture occurred at the same site, which was surgically treated with an endoprosthesis (Fig. 5B and C) on April 20, 2016. Eight years after surgery, metastases to the left second and tenth ribs (Fig. 6A and B) and multiple spinal metastases developed (Fig. 6C–E) on October 14, 2017. Ten years after the surgery, right pulmonary pleural effusion was observed (Fig. 7A) on September 5, 2020. The patient underwent 5 pleural fluid drainages and 1 pleurodesis by our respiratory physician and thoracic surgeon. However, despite these interventions, pleural effusion and lung metastases increased, resulting in respiratory distress. Pazopanib was discontinued, and the patient was transitioned to best support care (BSC). Pazopanib was continued until the initiation of BSC status. Eleven years after surgery, a thrombus appeared in the inferior vena cava (D-dimer value = 9.8) (Fig. 7B) on August 10, 2020; under the supervision of a cardiologist at our hospital, anticoagulant therapy was initiated, and the thrombus was reduced. However, 6 months later, at 11 years and 6 months postoperatively from the first surgery, the patient succumbed to the disease on February 1, 2021.

## 3. Discussion

Hemangiopericytomas are rare tumors of mesenchymal origin, accounting for <2% of all soft tissue tumors,^[9]^ and have been considered the same as SFT per the WHO classification since 2016.^[1]^ No notable sex difference in the incidence of SFT, which is more common in patients aged between 40 and 70 years,^[10]^ similar to the characteristics observed in this case. The similarities include the site of origin,^[1]^ patient age,^[1]^ presence of abundant blood vessels^[1,3]^ in the pathology, and the relatively slow growth rate of the tumor.^[1,2]^ An SFT can occur anywhere in the body.^[10]^ Typical sites are the pleura and surrounding areas.^[11]^ The most common extrathoracic sites are the retroperitoneum, deep soft tissues of the extremities, abdominal cavity, and head and neck, and 30% to 40% occur in deep soft tissues.

CD34 is the most consistent marker for SFT; however, some studies denied this.^[12]^ Genetic diagnosis identified the NAB2-STAT6 fusion gene.^[13]^ In this case, the patient tested positive for CD34; however, hemangiopericytoma and SFT were separately considered at the time of diagnosis, and genetic testing was not performed.

Extensive resection is the primary treatment for SFT.^[14]^ Owing to the rich vascularity of the tumor, intraoperative blood loss is high (average operating time, 165 minutes; average blood loss, 500 mL).^[14]^ In this case, the operative time was relatively short because marginal resection was performed, and the blood loss was average.

Radiotherapy is often used to treat malignant tumors with positive postoperative margins.^[15,16]^ Regarding the radiation dose, 50 Gy was the most effective and had the fewest side effects and complications.^[17]^ However, no solid evidence exists regarding the efficacy of RT regimens for SFT. In this case, the dose was 66 Gy; however, no complications occurred, and local recurrence was prevented. RT may be effective after marginal resection of SFT.^[18]^

At the time of diagnosis, both weekly PTX and pazopanib were reportedly effective against hemangiopericytomas.^[6,7]^ In the 2010s, chemotherapy with doxorubicin and ifosfamide was reported to be effective against SFT.^[19]^ The understanding of the involvement of the vascular endothelial growth factor (VEGF) and vascular endothelial growth factor receptor (VEGFR) pathways in the development of nongastrointestinal stromal tumor soft-tissue sarcomas, coupled with the discovery of the antitumor activity of agents inhibiting these pathways in other solid tumors, led to the development of anti-VEGF angiogenesis inhibitors in soft-tissue sarcomas. These include monoclonal antibodies (for example, bevacizumab) or small molecules (for example, anti-VEGFR tyrosine kinase inhibitors).^[8]^ After early-phase trials established optimal dosing and safety profiles and received approval for the treatment of advanced renal cell carcinoma, pazopanib was studied in STS. A landmark randomized phase III trial showed improved progression-free survival with pazopanib compared to placebo in patients with various subtypes of previously treated STS.^[20]^ Pazopanib has also shown efficacy in treating hemangiopericytomas, with evidence indicating an improved progression-free survival (PFS) of 4.7 months (95% confidence interval 0.7–9.6).^[21]^ Furthermore, recent evidence supports pazopanib as an effective treatment option in primary and secondary treatments for recurrent or metastatic SFT, with a PFS improvement of 6.2 months.^[22]^ The study revealed respective response rates of 0% and 50%, whereas the respective disease control rates were 88.9% and 75%.^[22]^ Additionally, the PFS was 6.2 months (95% confidence interval 3.2–8.8).^[22]^ Moreover, the treatment line and high frequency of mitosis were not predictive of PFS.^[2]^ Based on the evidence at the time of diagnosis, the patient was treated weekly with PTX and pazopanib. The volume of pazopanib was reduced to 1/4th of the standard dose because of concerns about pneumothorax, a known side effect of pazopanib, which was exacerbated by the existing pleural effusion. Despite the development of bone metastases, pazopanib treatment was continued due to the absence of viable alternative therapies and at the patient’s request. However, pazopanib did not prove effective in this case, and its impact on prolonging survival remains unclear. Pathological fractures occur in approximately 10% to 25% of patients with cancer.^[23]^ The most common site of pathological fractures is the proximal femur, which accounts for 75% of all fractures.^[24]^ Surgical treatment can be broadly divided into tumor arthroplasty and intramedullary nailing.^[25]^ In this case, tumor arthroplasty was performed to stabilize the affected limbs.

Risk factors for thrombosis in patients with cancer include inpatient treatment, history of blood clots, family history, chemotherapy, fever, and elevated C-reactive protein.^[26]^ Given that this patient had 3 risk factors – hospitalization, chemotherapy, and fever – for the development of thrombosis, it is crucial to monitor her for the development of thrombosis during and after treatment.

The local recurrence rate of SFT is reported to be 9% to 19%, and the distant metastasis rate is 0% to 36%.^[12,27,28]^ In addition, the 5-year survival rate is reported to be 70% to 84%, and the 10-year survival rate is 47%.^[12,27,28]^ Poor prognostic factors include age > 55 years, mitosis > 2/mm^2^, and tumor size > 5 cm.^[29]^ Despite the poor prognostic factors, the patient survived > 11 years after surgery and had a favorable outcome.

## 4. Conclusions

The treatment course for SFTs at our hospital yielded favorable outcomes. Oncologic surgeons must anticipate all possible complications associated with malignancies and be prepared with appropriate countermeasures. Specifically, if the margin is positive, 1 must be prepared for all possible complications arising due to malignancy, such as the need for postoperative radiation and its associated side effects, including pathological fractures and inevitable venous thrombosis that occurs when the patient is in the near-end stage of the disease and in a prolonged bedridden state.

## Acknowledgments

The authors would like to thank Editage (www.editage.jp) for the English language editing.

## Author contributions

**Conceptualization:** Kazuhiko Hashimoto, Shunji Nishimura, Ryosuke Kakinoki, Koji Goto.

**Data curation:** Kazuhiko Hashimoto, Shunji Nishimura, Tomohiko Ito, Koji Goto.

**Formal analysis:** Kazuhiko Hashimoto, Tomohiko Ito, Ryosuke Kakinoki.

**Investigation:** Kazuhiko Hashimoto, Shunji Nishimura, Tomohiko Ito, Ryosuke Kakinoki, Koji Goto.

**Methodology:** Kazuhiko Hashimoto, Shunji Nishimura, Tomohiko Ito, Koji Goto.

**Project administration:** Kazuhiko Hashimoto, Tomohiko Ito, Ryosuke Kakinoki, Koji Goto.

**Resources:** Kazuhiko Hashimoto, Tomohiko Ito, Ryosuke Kakinoki.

**Software:** Kazuhiko Hashimoto.

**Supervision:** Kazuhiko Hashimoto, Ryosuke Kakinoki.

**Validation:** Kazuhiko Hashimoto, Shunji Nishimura, Tomohiko Ito, Ryosuke Kakinoki, Koji Goto.

**Visualization:** Kazuhiko Hashimoto, Shunji Nishimura, Tomohiko Ito, Koji Goto.

**Writing – original draft:** Kazuhiko Hashimoto, Shunji Nishimura, Tomohiko Ito, Ryosuke Kakinoki, Koji Goto.

**Writing – review & editing:** Kazuhiko Hashimoto, Shunji Nishimura, Tomohiko Ito, Ryosuke Kakinoki, Koji Goto.
